# Supplementary material for: Complex‐centric proteome profiling by SEC‐SWATH‐MS
Source: Mol Syst Biol. 2019 Jan 14;15(1):e8438. doi: 10.15252/msb.20188438 (PMC6346213; doi:10.15252/msb.20188438)
Supplement: Supplementary file 8 — Dataset EV7 [file MSB-15-e8438-s008.zip › feature_plots_string/Q9BZJ0.pdf]

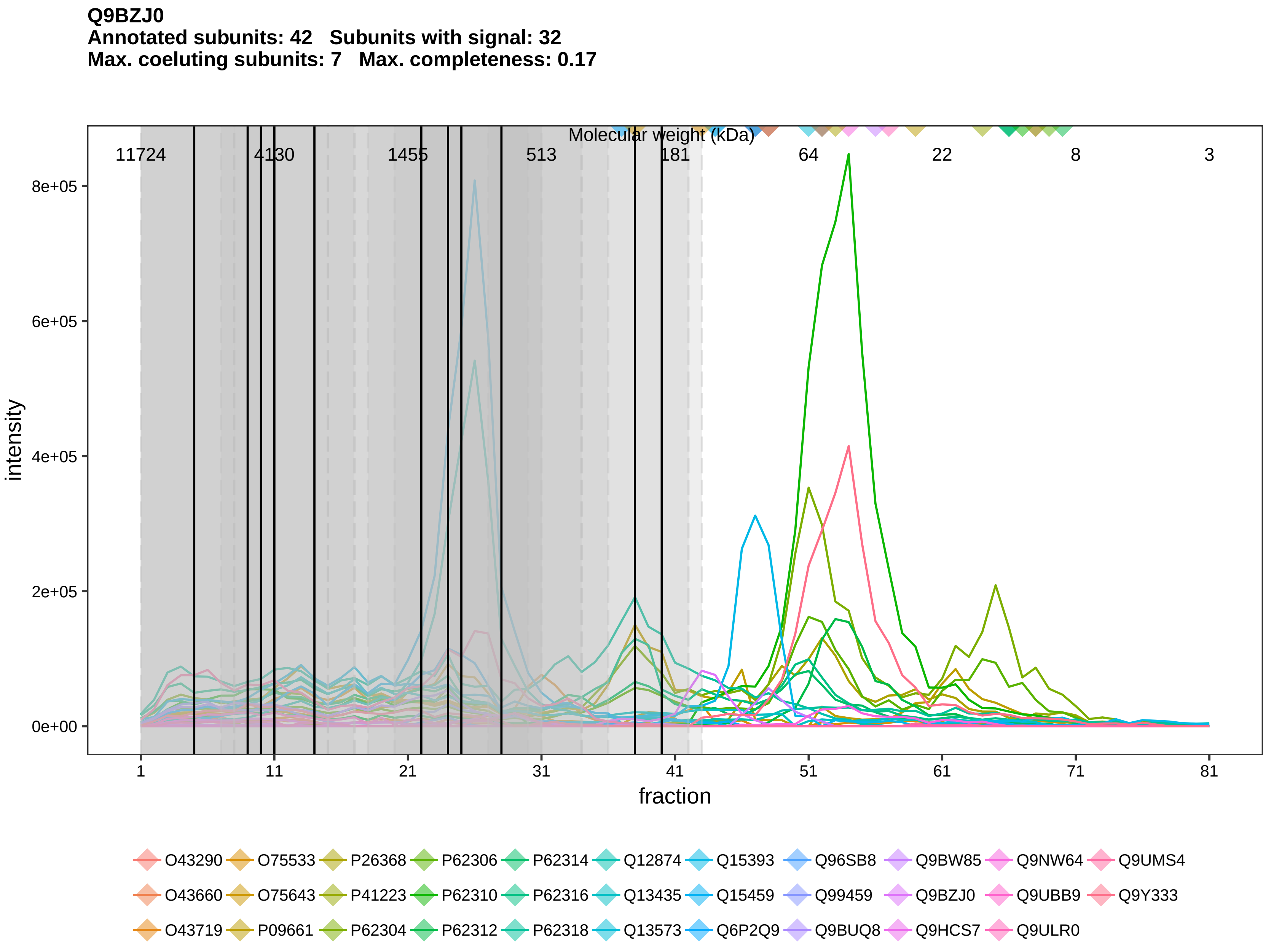

Q9BZJ0
Annotated subunits: 42 Subunits with signal: 32
Max. coeluting subunits: 7 Max. completeness: 0.17
Molecular weight (kDa)
11724
4130
1455
513
181
64
22
8
3
8e+05
6e+05
4e+05
2e+05
0e+00
intensity
1
11
21
31
41
51
61
71
81
fraction
O43290
O43660
O43719
O75533
O75643
P09661
P26368
P41223
P62304
P62306
P62310
P62312
P62314
P62316
P62318
Q12874
Q13435
Q13573
Q15393
Q15459
Q6P2Q9
Q96SB8
Q99459
Q9BUQ8
Q9BW85
Q9BZJ0
Q9HCS7
Q9NW64
Q9UBB9
Q9ULR0
Q9UMS4
Q9Y333
